# Can Lunchtime Teaching Help Deliver Psychotherapy Competencies? an Evaluation of a Case-Based Teaching Model in an Inpatient Psychiatric Setting

**DOI:** 10.1192/bjo.2026.11581

**Published:** 2026-06-30

**Authors:** Sean Treadwell

**Affiliations:** Leeds and York NHS Foundation Trust, Leeds, United Kingdom

## Abstract

**Aims:**

**Background**

Psychotherapy competencies form a core part of psychiatry training, yet access to psychotherapy-related learning opportunities is often limited, particularly within inpatient psychiatric settings. Trainees and students working on inpatient wards may have fewer opportunities to observe or practise psychologically informed approaches, despite the centrality of formulation, therapeutic relationships, and reflective practice in this context. Teaching models that offer exposure to applied psychotherapy-related skills across varied training grades may help address this gap in everyday inpatient care.

**Aims:** To evaluate a regular lunchtime, case-based discussion group designed to support the development of psychotherapy-related core psychiatry training competencies, and to explore participants’ perceptions of its usefulness, educational impact, and influence on engagement with psychiatry as a specialty.

**Methods:**

A fortnightly 50-minute lunchtime case-based discussion group was established at an inpatient psychiatric hospital in Leeds and was open to medical students and resident doctors of mixed grades. Sessions were centred on cases brought by participants, with facilitated discussion focusing on psychological formulation, therapeutic relationships, and the application of psychotherapy-related concepts to inpatient clinical work. Anonymous post-session feedback was collected using Likert-scale measures assessing perceived usefulness, impact on engagement with psychiatry, and influence on interest in psychiatry as a specialty. Participants also indicated which psychotherapy-related core psychiatry curriculum key capabilities they felt were addressed during the group. Data were analysed descriptively.

**Results:**

Across four fortnightly groups, a total of 13 participants completed feedback. Mean ratings were high across all measured domains, with an average score of 5.8 for perceived usefulness, 5.7 for increased engagement in psychiatry, and 5.4 for increased interest in psychiatry as a specialty (Likert scale 1–6). Of the 16 psychotherapy-related core psychiatry curriculum key capabilities listed, participants reported that an average of 10 were met per group, indicating broad perceived alignment with curriculum requirements.

**Conclusion:**

This evaluation suggests that a regular, lunchtime, case-based discussion group may offer a feasible and acceptable way of supporting the development of psychotherapy-related competencies within an inpatient psychiatric setting. By providing trainees and students of varied grades with structured exposure to applied psychotherapy-related skills in the context of inpatient work, such groups may help bridge the gap between formal psychotherapytraining and everyday clinical practice. As a low-resource and inclusive teaching model, this approach may usefully complement existing training pathways.

